# A Blueberry-Enriched Diet Attenuates Nephropathy in a Rat Model of Hypertension via Reduction in Oxidative Stress

**DOI:** 10.1371/journal.pone.0024028

**Published:** 2011-09-15

**Authors:** Carrie M. Elks, Scott D. Reed, Nithya Mariappan, Barbara Shukitt-Hale, James A. Joseph, Donald K. Ingram, Joseph Francis

**Affiliations:** 1 Comparative Biomedical Sciences, Louisiana State University School of Veterinary Medicine, Baton Rouge, Louisiana, United States of America; 2 Neurosignaling Laboratory, Pennington Biomedical Research Center, Louisiana State University System, Baton Rouge, Louisiana, United States of America; 3 United States Department of Agriculture-Agriculture Research Services, Human Nutrition Research Center on Aging, Tufts University, Boston, Massachusetts, United States of America; 4 Nutritional Neuroscience and Aging Laboratory, Pennington Biomedical Research Center, Louisiana State University System, Baton Rouge, Louisiana, United States of America; L' Istituto di Biomedicina ed Immunologia Molecolare, Consiglio Nazionale delle Ricerche, Italy

## Abstract

**Objective and Background:**

To assess renoprotective effects of a blueberry-enriched diet in a rat model of hypertension. Oxidative stress (OS) appears to be involved in the development of hypertension and related renal injury. Pharmacological antioxidants can attenuate hypertension and hypertension-induced renal injury; however, attention has shifted recently to the therapeutic potential of natural products as antioxidants. Blueberries (BB) have among the highest antioxidant capacities of fruits and vegetables.

**Methods and Results:**

Male spontaneously hypertensive rats received a BB-enriched diet (2% w/w) or an isocaloric control diet for 6 or 12 weeks or 2 days. Compared to controls, rats fed BB-enriched diet for 6 or 12 weeks exhibited lower blood pressure, improved glomerular filtration rate, and decreased renovascular resistance. As measured by electron paramagnetic resonance spectroscopy, significant decreases in total reactive oxygen species (ROS), peroxynitrite, and superoxide production rates were observed in kidney tissues in rats on long-term dietary treatment, consistent with reduced pathology and improved function. Additionally, measures of antioxidant status improved; specifically, renal glutathione and catalase activities increased markedly. Contrasted to these observations indicating reduced OS in the BB group after long-term feeding, similar measurements made in rats fed the same diet for only 2 days yielded evidence of increased OS; specifically, significant increases in total ROS, peroxynitrite, and superoxide production rates in all tissues (kidney, brain, and liver) assayed in BB-fed rats. These results were evidence of “hormesis” during brief exposure, which dissipated with time as indicated by enhanced levels of catalase in heart and liver of BB group.

**Conclusion:**

Long-term feeding of BB-enriched diet lowered blood pressure, preserved renal hemodynamics, and improved redox status in kidneys of hypertensive rats and concomitantly demonstrated the potential to delay or attenuate development of hypertension-induced renal injury, and these effects appear to be mediated by a short-term hormetic response.

## Introduction

Oxidative stress produced by overproduction of reactive oxygen species/reactive nitrogen species (RONS) or inefficient antioxidant defenses appears to be involved in the development and progression of hypertension and hypertension-induced renal injury [1], [2]. The detrimental role of RONS in hypertension-induced renal injury has fostered an increased interest in the therapeutic potential of antioxidants; however, the majority of studies thus far have employed synthetic antioxidants to prevent or attenuate the detrimental effects of ROS both *in vivo* and *in vitro*. Recently, attention has been directed to natural products as sources of antioxidants [3]. Most plant cells contain antioxidant mechanisms to detoxify free radicals, which are produced during normal cellular metabolic processes [4]. In particular, small berry fruits have been demonstrated to have high contents of several antioxidant compounds, including anthocyanins and phenolics. These metabolites function to protect plants against photodynamic reactions by quenching ROS, and have been suggested to have protective effects against several human diseases [5], [6].

Blueberries (BB; *Vaccinium spp.*) have among the highest antioxidant capacities of fruits and vegetables tested to date, and contain polyphenols such as anthocyanins, proanthocyanidins, and phenolic acids, and flavanols [7]. BB-enriched diets and BB extracts have been shown to attenuate and even improve age-related behavioral and neuronal deficits in rodents [8]–[11]. BB supplementation can also attenuate proinflammatory cytokine production in rat glial cells [12] and protect the rat heart from ischemia [13]. Additionally, hypertensive rats on BB-supplemented diets exhibit significantly lower systolic and mean arterial pressures and renal nitrite content [14]. Therefore, it is plausible to suggest that dietary BB supplementation may have a tissue-protective effect in various pathologic conditions. In this light, the general objective of the current study was to assess the chronic effects of a BB-enriched diet on blood pressure (BP) and renal hemodynamics in a rat model of hypertension-induced renal injury. The hypothesis was that the dietary BB supplementation would reduce oxidative stress and thus attenuate renal damage. As an extension of this hypothesis, rats were also subjected to a short-term (2-day) exposure of the BB-supplemented diet to determine whether a hormetic effect would be observed. Hormesis has been proposed as the mechanism mediating the protective effects of many plant products [15], [16]. Specifically, the hypothesis was that during short-term exposure, increased RONS production would be observed which would lead to up-regulation of antioxidant defense mechanisms to enhance long-term protection.

## Materials and Methods

### Ethics Statement

All experimental procedures were in compliance with all applicable principles set forth in the National Institutes of Health Guide for the Care and Use of Laboratory Animals (Publication No.. 85-23, revised 1996). This study was approved by the Institutional Animal Care and Use Committee of the Louisiana State University School of Veterinary Medicine (protocol approval number 09-008).

### Experimental Design

#### Experiment 1: Chronic feeding studies

Forty-eight male stroke-prone spontaneously hypertensive rats (SHRSP) and thirty-two male normotensive Wistar-Kyoto (WKY) rats were used for chronic feeding studies. Rats were 7 weeks old with baseline body weights between 130 and 150 grams. Rats were randomly divided into four diet groups for each chronic study: WKY control (WC), WKY blueberry (WBB), SHRSP control (SC), or SHRSP BB (SBB). Animals were fed control or BB-enriched diets for 6 weeks or 12 weeks. All animals in all groups were provided 1% sodium chloride in tap water for the duration of both chronic studies. All animals were subjected to acute determination of glomerular filtration rate and renal plasma flow, as previously described [17], at the end of the 6-week or 12-week feeding periods. Rats were euthanized, kidneys were excised, and cortex and medulla separated for analyses. Kidneys were formalin-fixed, paraffin-embedded, and then sectioned (3 um); sections were placed on slides and stained with Masson's Trichrome for evaluation by a veterinary pathologist who was blinded to experimental conditions.

#### Experiment 2: Short-term feeding studies in SHRSP rats

For short-term feeding studies to evaluate hormetic effects, 24 additional 7-week-old male SHRSP were used. Rats (n = 12 in each group) were fed control or BB-enriched diets for 2 days. Rats were euthanized after which heart, brain, kidney, and liver tissues were collected. In both 2-day and chronic studies, fresh tissues were used for electron paramagnetic resonance (EPR) spectroscopy studies, and frozen tissues were used for antioxidant studies.

### Diets

Diets were prepared by Harlan Teklad (Madison, WI) using a reformulated NIH-31 diet by adding 20 g/kg lyophilized BB or 20 g/kg dried corn. To prepare the 2% BB diet, the berries were homogenized in water, centrifuged, lyophilized and added to the NIH-31 rodent chow. The amount of corn in the control diet was adjusted to compensate for the added volume of BB, in order to make the two diets isocaloric [18]. Food consumption was measured weekly for the chronic feeding studies by weighing feed before placing it in each cage, and subtracting the weight of remaining feed at the end of each week. Rats maintained on BB diets for 6 weeks consumed an average of 371 mg/day (WBB) or 374 mg/day (SBB) of lyophilized blueberries, roughly equivalent to 4.1 g/day or 4.2 g/day, respectively, of fresh blueberries. Rats maintained on BB diets for 12 weeks consumed an average of 397 mg/day (WBB) or 399 mg/day (SBB) of lyophilized blueberries, roughly equivalent to 4.4 g/day or 4.5 g/day, respectively, of fresh blueberries.

### Blood pressure measurements

In rats from all chronic feeding groups, tail blood pressures (BP) were measured noninvasively using a Coda 6 Volume-Pressure Recording System (Kent Scientific, Torrington, CT), as previously described [1], [19]. Briefly, eight unanesthetized rats from each group were warmed to an ambient temperature of 30°C by placing them in a holding device mounted on a thermostatically controlled warming plate. Tail cuffs were placed on animals, and animals were allowed to acclimate to cuffs for 10 minutes prior to each pressure recording session. All animals were habituated to the blood pressure system and to the holders daily for one week prior to the initiation of experimental measurements. All measurements were taken within the same 2-hour time window each day. Each session consisted of 30 cycles. BP was measured on five consecutive days each week, and values were averaged from at least six consecutive cycles. BP was measured at baseline (7 weeks of age) and then weekly until the end of either chronic study period.

### Acute renal clearance experiments

Nine rats from each 6-week feeding group and nine rats from each 12-week feeding group were subjected to renal clearance experiments at the end of their respective feeding periods as previously described [1]. Briefly, each rat was anesthetized with Inactin (thiobutabarbital; 100 mg/kg), the right inguinal area was shaved, a small (<2 cm) incision made, and femoral vessels isolated. The right femoral artery was cannulated with heparin-primed (100 U/ml) PE-50 polyethylene tubing connected to a pressure transducer (PowerLab data acquisition system; ADInstruments, Colorado Springs, CO) for continuous measurement of arterial pressure. The right femoral vein was catheterized with heparin-primed PE-50 tubing for infusion of solutions at 20 µl/min. An isotonic saline solution containing 6% albumin was infused during surgery. After surgery, the infusion fluid was changed to isotonic saline containing 2% bovine serum albumin (BSA), 7.5% inulin (Inutest), and 1.5% PAH, and a 300 ul bolus of this solution was administered at the start of each clearance experiment. The bladder was exposed via a suprapubic incision and catheterized with a PE-200 tube (with one end flanged) for gravimetric urine collection. After a 15- to 20-minute stabilization period, a 30-minute clearance period was conducted to assess values of renal hemodynamic parameters. An arterial blood sample was collected at the end of the 30-minute clearance collection period for measurement of plasma inulin and PAH concentrations. Plasma inulin and PAH concentrations were measured colorimetrically to determine glomerular filtration rate (GFR) and renal plasma flow (RPF), respectively.

### Electron paramagnetic resonance (EPR) spectroscopy

Total ROS, superoxide, and peroxynitrite production rates were measured in pieces of kidney cortex or medulla (chronic and 2-day feeding studies) and in liver and cerebral cortex (2-day feeding study) via EPR spectroscopy as previously described [1], [19]–[24]. In this EPR protocol, ‘total ROS’ represents all reactive oxygen species; however, the major sources trapped by the spin trap used are superoxide, hydrogen peroxide, and hydroxyl radical, with other species as minimal contributors. Briefly, tissue pieces were incubated at 37°C with 6.6 µl of CMH (200 µM) for 30 minutes for ROS measurement; 1.5 µl of PEG-SOD (50 U/µl) for 30 minutes, then CMH for an additional 30 minutes for superoxide measurement; or 30 µl of CPH (500 µM) for 30 minutes for peroxynitrite measurement. Aliquots of incubated probe media were then taken in 50 µl disposable glass capillary tubes (Noxygen Science Transfer and Diagnostics, Elzach, Germany) for determination of total ROS, superoxide, or peroxynitrite production, under previously established EPR settings.

### Measurement of renal catalase and glutathione levels

Catalase activity and total glutathione (GSH) levels were measured in kidney cortex and medulla (chronic and 2-day feeding studies) and also in liver and left ventricle (2-day feeding study) using commercially available kits (Cayman Chemical, Ann Arbor, MI) according to manufacturer's instructions, as previously described [1],[25].

### Measurement of urine creatinine levels

Creatinine was quantified in urine with a QuantiChrom Creatinine kit (BioAssay Systems, Hayward, CA) according to manufacturer's instructions, as previously described [17].

### Measurement of urine and tissue nitrate/nitrite levels

Total nitrate/nitrite levels in kidney cortex and medullary tissues and in urine were quantified with a Nitrate/Nitrite Colorimetric Assay kit (Cayman Chemical, Ann Arbor, MI), according to manufacturer's instructions.

### Statistical analyses

A two-way ANOVA (strain x diet) was used to analyze blood pressure, food consumption, body weight, physiological, biochemical, and EPR data at each time-point. Where significant main effects or interactions were found, individual planned comparisons were made using Student's t-tests for all other chronic feeding study data specifically to compare WC and WBB animals; WC and SC animals; and SC and SBB animals. T-tests were also used to compare results from SHR C and SHR BB groups for the 2-day feeding study. In all cases, p≤0.05 was accepted as the level of statistical significance.

## Results

### A. Chronic feeding studies

#### Body weight and food intake

Consistent with past studies using similar dietary formulations [8],[13],[14],[26], weekly food consumption and body weight gain did not differ among any of the diet groups in the chronic feeding studies. Mean starting body weights in the WKY and SHR animals at baseline before assignment to groups were 138±3 g and 142±2 g, respectively; at the end of the 6 week study, the mean body weights were as follows: WC = 252±9 g; WBB = 246±3 g; SC = 250±3 g; and SBB  =  252±3 g. At the end of the 12 week study, mean body weights were as follows: WC  =  346±4 g; WBB = 342±5 g; SC = 334±6 g; and SBB = 333±8 g.

#### Effect of chronic BB feeding on blood pressures and renal hemodynamic parameters

**Figure 1:**
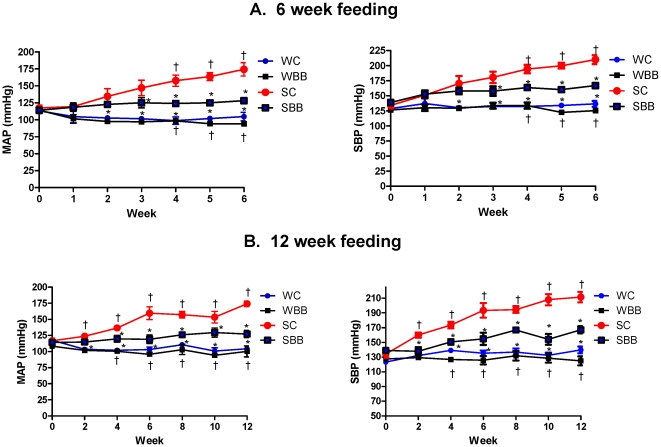
Blueberry-enriched diet delays the progression of hypertension. Mean arterial and systolic blood pressures were assessed in rats fed a control diet or a blueberry-enriched diet for 6 weeks (A) or 12 weeks (B). ^*^ p<0.05 vs. SC; ^†^ p<0.05 vs. SBB.

Figure 1 presents the BP trends for each group of rats in both the 6-week and 12-week studies. Compared to SC rats, the mean arterial and systolic pressures of the SBB rats were significantly lower by the second week of the 6-week and 12-week studies, and remained significantly lower for the remainders of both chronic studies.

Table 1 presents the renoprotective effects of BB diet in SHRSP rats fed for 6 weeks or 12 weeks. Glomerular filtration rate and renal blood flow were higher, and renal vascular resistance was lower, in 6-week and 12-week SBB rats when compared to SC rats. There were no significant differences in renal hemodynamic or BP measures between WC or WBB animals.

**Table 1:** Renal hemodynamic indices in control- or blueberry-fed rats after 6 weeks or 12 weeks of feeding.

		WC (n = 9)	WBB (n = 9)	SC (n = 9)	SBB (n = 9)
**6 WEEKS**	**GFR** (ml/min/g KW)	0.95±0.05#	0.92±0.05	0.59±0.04* $	0.97±0.07#
	**RBF** (ml/min/g KW)	7.98±0.25#	8.42±0.32	5.96±0.35* $	7.71±0.17#
	**RVR** (mmHg/ml/min/g KW)	13.33±0.70#	14.28±1.24	28.04±1.39* $	13.34±0.63#
	**Urine Cr** (mg/dl)	161.2±7.7#	142.3±12.9	70.1±4.8* $	121.5±8.2#
	**FE_Na_** (%)	0.28±0.02#	0.32±0.03	0.59±0.04* $	0.38±0.04#
	**KW/BW** (mg/g)	3.68±0.04#	3.70±0.04	4.73±0.05*	4.72±0.06
**12 WEEKS**	**GFR**	0.90±0.06#	1.11±0.09	0.53±0.04* $	1.02±0.07#
	**RBF**	7.03±0.25#	8.42±0.75	3.62±0.22* $	6.80±0.59#
	**RVR**	15.98±1.14#	14.05±1.41	36.71±2.10* $	15.49±1.22#
	**Urine Cr**	119.0±9.0#	119.8±12.3	66.6±4.2* $	129.1±12.2#
	**FE_Na_**	0.36±0.02#	0.34±0.03	0.67±0.03* $	0.46±0.04#
	**KW/BW**	3.13±0.05#	3.14±0.06	4.98±0.15* $	4.48±0.05#

Abbreviations used: WC  =  WKY corn-fed, WBB  =  WKY blueberry-fed, SC  =  SHRSP corn fed, SBB  =  SHRSP blueberry-fed, GFR  =  glomerular filtration rate, RBF  =  renal blood flow, RVR  =  renal vascular resistance, KW  =  kidney weight, Cr  =  creatinine, FE_Na_  =  fractional excretion of sodium.

*p≤0.05 vs. WC;

# p≤0.05 vs. SC;

$ p≤0.05 vs. SBB.

#### Effect of chronic BB feeding on cortical and medullary free radical production rates

The decreases in total ROS, superoxide, and peroxynitrite seen with chronic BB feeding for 6 or 12 weeks appear in Table 2. For both 6- and 12-week studies, SBB rats exhibited significantly lower free radical production rates than SC rats.

**Table 2:** Total ROS, superoxide, and peroxynitrite production rates as measured by EPR in tissues of control- or blueberry-fed rats after 6 or 12 weeks of feeding.

	WC (n = 8-10)	WBB (n = 8-10)	SC (n = 8-10)	SBB (n = 8-10)
	**KIDNEY CORTEX**			
**Total ROS** (uM/mg protein/minute)				
**6 weeks**	0.067±0.012#	0.099±0.005$	0.199±0.027* $	0.069±0.011#
**12 weeks**	0.115±0.013#	0.112±0.009$	0.429±0.038* $	0.195±0.026#
**Superoxide** (uM/mg protein/minute)				
**6 weeks**	0.040±0.014#	0.028±0.006	0.136±0.026* $	0.030±0.007#
**12 weeks**	0.067±0.018#	0.063±0.013	0.165±0.013* $	0.063±0.006#
**Peroxynitrite** (uM/mg protein/minute)				
**6 weeks**	0.017±0.002#	0.022±0.003$	0.053±0.011* $	0.011±0.003#
**12 weeks**	0.020±0.006#	0.028±0.004	0.111±0.021* $	0.028±0.020#
	**KIDNEY MEDULLA**			
**Total ROS**				
**6 weeks**	0.057±0.011#	0.087±0.007$	0.188±0.025* $	0.118±0.010#
**12 weeks**	0.157±0.014#	0.151±0.018	0.314±0.017* $	0.170±0.017#
**Superoxide**				
**6 weeks**	0.056±0.011#	0.051±0.013	0.122±0.009* $	0.048±0.011#
**12 weeks**	0.079±0.012#	0.085±0.008	0.194±0.017* $	0.089±0.019#
**Peroxynitrite**				
**6 weeks**	0.027±0.006#	0.021±0.005	0.050±0.005* $	0.025±0.005#
**12 weeks**	0.052±0.017#	0.065±0.005$	0.128±0.009* $	0.089±0.019#

Abbreviations used: WC  =  WKY corn-fed, WBB  =  WKY blueberry-fed, SC  =  SHRSP corn fed, SBB  =  SHRSP blueberry-fed, ROS  =  reactive oxygen species.

*p≤0.05 vs. WC;

# p≤0.05 vs. SC;

$ p≤0.05 vs. SBB.

#### Effect of chronic BB feeding on catalase and glutathione activities

**Figure 2:**
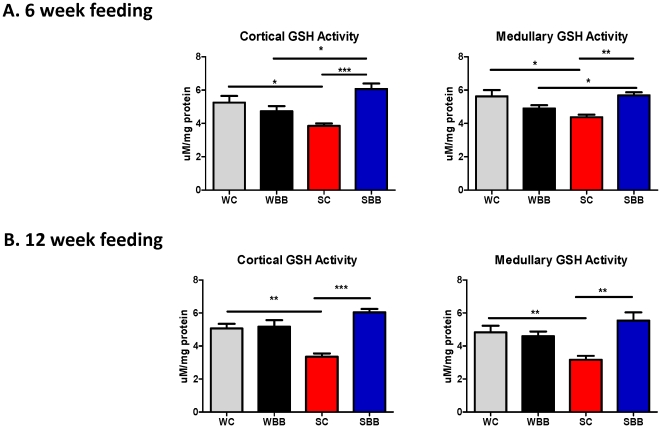
Blueberry-enriched diet improves glutathione activity in hypertensive rats. Glutathione activities were assessed in renal cortical and medullary tissues of rats fed a control diet or a blueberry-enriched diet for 6 weeks (A) or 12 weeks (B). ^*^p<0.05; ^**^ p<0.01; ^***^ p<0.001.

**Figure 3:**
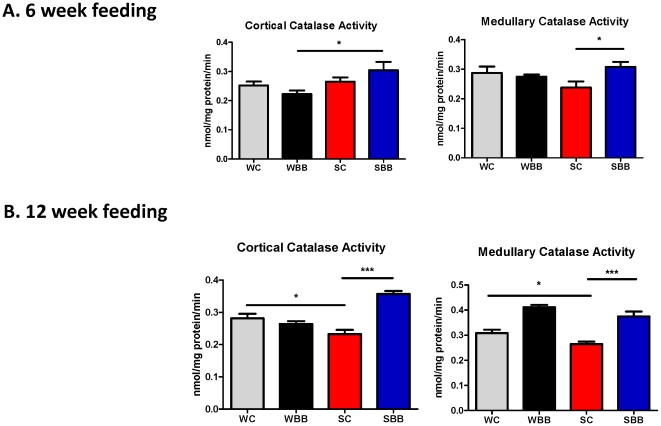
Blueberry-enriched diet improves catalase activity in hypertensive rats. Catalase activities were assessed in renal cortical and medullary tissues of rats fed a control diet or a blueberry-enriched diet for 6 weeks (A) or 12 weeks (B). ^*^p<0.05; ^**^ p<0.01; ^***^ p<0.001.

The increases in cortical and medullary catalase and glutathione activities noted with chronic BB-enriched diet feedings appear in Figures 2 and 3, respectively. For both chronic feeding studies, SBB rats exhibited significantly higher catalase and glutathione activities than SC rats. There were no differences in antioxidant activities between WC and WBB rats at the conclusion of the 6- or 12-week studies.

#### Effect of chronic BB feeding on renal pathology

**Figure 4:**
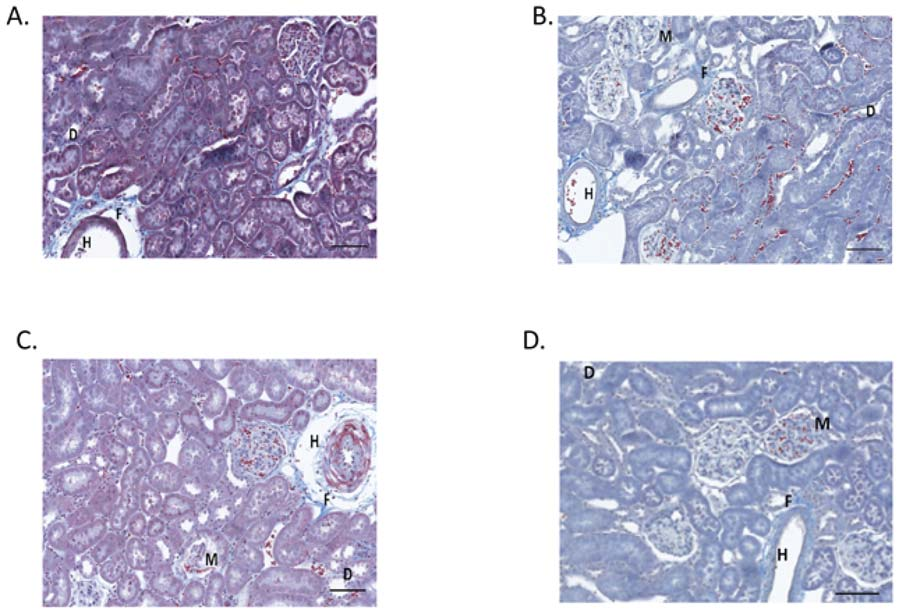
Blueberry-enriched diet improves renal pathology in hypertensive rats fed for 6 weeks. Trichrome-stained kidney sections were evaluated by a veterinary pathologist who was blinded to experimental conditions. Kidneys from (A) WC and (B) WBB rats exhibited similar histological appearance, with mild pathologic changes. Kidneys from (C) SC rats exhibited greater incidence and severity of pathologic change, while kidneys from (D) SBB rats exhibited very little pathologic change. Scale bar  =  200 um. F  =  fibrosis, H  =  vascular smooth muscle hypertrophy, D  =  tubular degeneration and ectasia, and M  =  glomerular parietal metaplasia.

**Figure 5:**
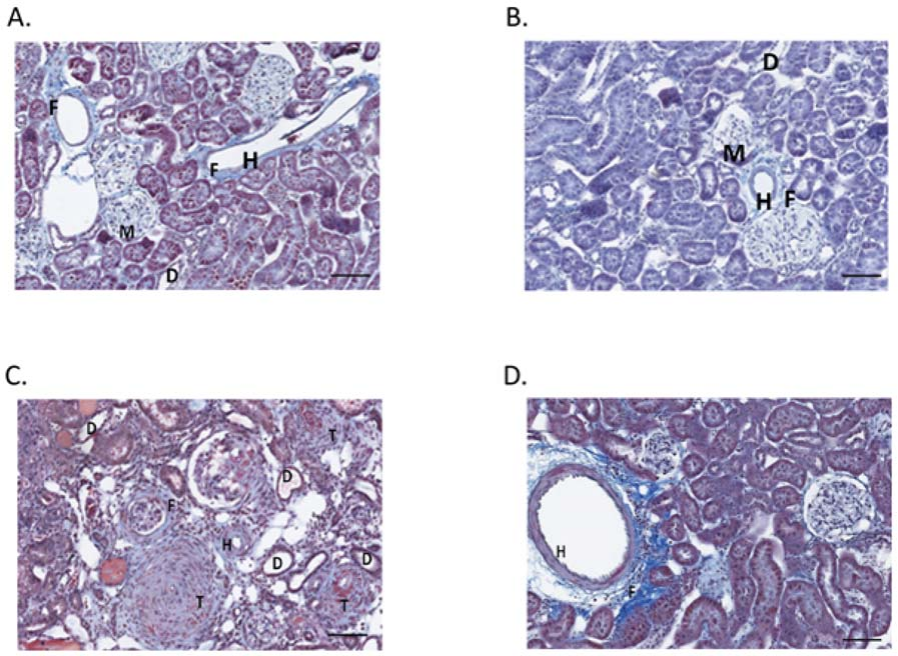
Blueberry-enriched diet improves renal pathology in hypertensive rats fed for 12 weeks. Trichrome-stained kidney sections were evaluated by a veterinary pathologist who was blinded to experimental conditions. In all four 12-week groups, the amount of interstitial, periarterial, and periglomerular fibrosis was greater than at six weeks. The SC rat kidneys (C) had severe arterial changes, with evidence of hypoxia. Kidneys from the SC rats also had the most severe fibrosis, arterial myointimal hyperplasia, tubular degeneration and necrosis, and evidence of hemoglobin and proteinaceous casts. The SBB rat kidneys (D) did exhibit arterial myointimal hyperplasia, though it was much less severe. Kidneys of WC (A) and WBB (B) rats fed for 12 weeks exhibited much less severe changes, but showed the same trends. Scale bar  =  200 um. F  =  fibrosis, H  =  vascular smooth muscle hypertrophy, T  =  organizing thrombus, D  =  tubular degeneration and ectasia, and M  =  glomerular parietal metaplasia.

Representative photomicrographs of kidneys from each 6-week and 12-week rat group appear in Figures 4 and 5, respectively. The kidneys of WC and WBB rats fed for 6 weeks exhibited similar appearances histologically. Kidneys of WC and WBB rats exhibited mild to moderate periarterial fibrosis, occasional tubular degeneration and dilation, mild glomerular parietal metaplasia, and minimal arterial hyperplasia. The kidneys of SBB rats fed for 6 weeks had relatively little renal pathologic change, with mild glomerular parietal metaplasia, occasional tubular degeneration and dilation and minimal periarterial fibrosis. The kidneys of SC rats, however, fed for 6 weeks had greater incidences of glomerular parietal metaplasia, tubular degeneration and dilation, and periarterial, interstitial, and periglomerular fibrosis, along with moderate to marked arterial myointimal hyperplasia.

In the kidneys of rats fed for 12 weeks, the amount of interstitial, periarterial, and periglomerular fibrosis was greater in all four groups than at six weeks. The SC rat kidneys had severe arterial changes which often were attended by evidence of renal hypoxia (tubular degeneration and necrosis). Arteries were frequently thrombosed and often had evidence of recanalization. The 12 week SC rat kidneys also had the most severe fibrosis, arterial myointimal hyperplasia, tubular degeneration and necrosis, and evidence of hemoglobin and proteinaceous casts. Though much less severe, the SBB rat kidneys did exhibit arterial myointimal hyperplasia with occasional mononuclear inflammatory cells present in the interstitium surrounding arteries. Kidneys of WC and WBB rats fed for 12 weeks exhibited much less severe changes, but showed the same trends.

### B. Short-term feeding studies in SHRSP rats

#### Body weights

No significant differences were found in body weights between control- or BB-diet fed SHRSP rats in the 2-day feeding study. Ending body weights were 161±5 g for rats fed the control diet and 163±5 g for rats fed the BB diet.

#### Effect of short-term BB feeding on cortical and medullary free radical production rates

As noted in Table 3, significant increases in production rates of total ROS, superoxide, and peroxynitrite were observed in cerebral cortex, liver, kidney cortex, and kidney medulla of SHRSP rats fed a BB diet for 2 days compared to those on control diet.

**Table 3:** Total ROS, superoxide, and peroxynitrite production rates as measured by EPR in tissues of control- or blueberry-fed rats after 2 days of feeding.

	Corn (n = 7)	Blueberry (n = 7)	P
**CEREBRAL CORTEX**			
** Total ROS**	0.126±0.008	0.156±0.015	0.0417
** Superoxide**	0.024±0.003	0.039±0.005	0.0087
** Peroxynitrite**	0.005±0.001	0.022±0.003	0.0004
**LIVER**			
** Total ROS**	0.371±0.026	0.530±0.057	0.0129
** Superoxide**	0.150±0.007	0.246±0.023	0.0046
** Peroxynitrite**	0.014±0.002	0.042±0.007	0.0069
**KIDNEY CORTEX**			
** Total ROS**	0.140±0.013	0.210±0.022	0.0289
** Superoxide**	0.041±0.005	0.063±0.004	0.0275
** Peroxynitrite**	0.015±0.002	0.034±0.004	0.0044
**KIDNEY MEDULLA**			
** Total ROS**	0.146±0.011	0.189±0.019	0.0136
** Superoxide**	0.060±0.011	0.106±0.025	0.0258
** Peroxynitrite**	0.035±0.006	0.075±0.011	0.0158

Abbreviations used: WC  =  WKY corn-fed, WBB  =  WKY blueberry-fed, SC  =  SHRSP corn fed, SBB  =  SHRSP blueberry-fed, ROS  =  reactive oxygen species.

#### Effect of short-term BB feeding on catalase and glutathione levels

Table 4 depicts the catalase and glutathione levels recorded in heart, liver, and kidney tissues of 2-day BB-fed SHRSP when compared to control diet-fed SHRSP. In the left ventricle and kidney cortex of SHRSP fed BB-diet for 2 days, catalase levels were increased when compared to those of SHRSP fed a control diet for 2 days. However, total GSH levels were lower in the left ventricle of 2 day BB-fed rats when compared to 2-day control diet-fed rats, with no significant changes in GSH levels noted in other tissues assayed.

**Table 4:** Catalase and total glutathione (GSH) levels as measured by colorimetric assays in tissues of control- or blueberry-fed rats after 2 days of feeding.

	Corn (n = 8-10)	Blueberry (n = 8-10)	P
**LEFT VENTRICLE**			
** Catalase**	0.293±0.019	0.373±0.022	0.0285
** Total GSH**	2.68±0.243	1.89±0.213	0.0495
**LIVER**			
** Catalase**	0.287±0.011	0.276±0.016	n.s.
** Total GSH**	1.91±0.159	1.85±0.065	n.s.
**KIDNEY CORTEX**			
** Catalase**	0.246±0.012	0.306±0.020	0.0331
** Total GSH**	2.11±0.187	2.23±0.091	n.s.
**KIDNEY MEDULLA**			
** Catalase**	0.325±0.010	0.310±0.028	n.s.
** Total GSH**	2.23±0.207	2.17±0.065	n.s.

Abbreviations used: WC  =  WKY corn-fed, WBB  =  WKY blueberry-fed, SC  =  SHRSP corn fed, SBB  =  SHRSP blueberry-fed, GSH  =  glutathione.

## Discussion

Primary (essential) hypertension remains a major cause of morbidity and mortality in Western society, and continues to be a leading cause of heart and kidney diseases [27]. The cause(s) of primary hypertension remain elusive; however, oxidative stress and proinflammatory cytokine production are known contributors [28],[29]. Nephropathy resulting from hypertension is the second leading cause of end-stage renal disease in the United States [27]; therefore, the most effective way to avoid the development of hypertensive nephropathy is to prevent hypertension or to delay its progression. In many cases, hypertension can be attenuated with pharmacological treatments including, but not limited to: diuretics, beta receptor antagonists, angiotensin converting enzyme antagonists, and angiotensin II receptor antagonists; however, these commonly used anti-hypertensives can also have undesirable side effects. Therefore, it is valuable to consider natural products, such as foods, as potentially therapeutic sources of antioxidants for a variety of conditions.

Thus far, a variety of pharmacotherapies have proven successful in decreasing renal damage in hypertensive animals; however, the possible benefits of dietary interventions have only recently come into focus. BB-enriched diets have been shown to decrease renal nitrite content, protect the myocardium from ischemia, and correct neurological deficits in rats [8],[10]–[14]. In the present study, we show for the first time that regular dietary supplementation with blueberries in SHRSP rats preserves renal hemodynamics and prevents oxidative stress in the kidney. We also demonstrate that BB may act via a hormetic mechanism in preventing long-term oxidative stress in the SHRSP rat.

After 6 weeks and 12 weeks of BB feeding, GFR and RBF measures were higher, estimated RVR was lower, renal free radical production was attenuated, and renal catalase and glutathione levels were preserved in BB-fed SHRSP when compared to those of SHRSP maintained on a control diet. The results of our chronic feeding experiments also demonstrate that total ROS, superoxide, and peroxynitrite production rates were significantly lower and antioxidant activities were significantly higher in BB-fed SHRSP than in corn-fed SHRSP. These results clearly demonstrate a protective antioxidant effect of BB feeding. The imbalance between superoxide production and NO production in the kidney is a primary contributor to renal oxidative stress and salt-sensitive hypertension [30],[31]. Oxidative stress is further enhanced in the kidneys of SHRSP that are salt-loaded (as were the SHRSP in this study) [31],[32]. We demonstrate here that the BB diet protected against oxidative renal damage by attenuating free radical production and preserving catalase and glutathione levels, and thereby improving BP and renal hemodynamics.

A possible mechanism for this renoprotection may be the scavenging of superoxide in kidney tissues, which has been shown to lower BP in various models of hypertension [31],[33]. BB are known scavengers of RONS, including superoxide, *in vitro* [34]. In further support of a renal superoxide scavenging mechanism, we found that the cortical and medullary production rates of peroxynitrite in BB-fed rats from both 6- and 12-week time-points were significantly lower compared to rats fed the control diet, as were urinary nitrate/nitrite and tissue nitrate/nitrite levels (Table 5). Further study is needed to determine conclusively whether this effect is responsible for the renoprotection afforded by chronic BB feeding. These chronic feeding studies were not intended to analyze specific signaling pathways responsible for preservation of renal hemodynamics and/or reduction of oxidative stress in the kidneys of hypertensive animals on a BB-enriched diet, but rather as a proof of concept. One assertion that can be made on the basis of these findings is that alterations in signaling were likely associated with decreases in RONS production and improvements in RONS scavenging.

**Table 5:** Urine and tissue nitrate/nitrite levels as measured by colorimetric assay in control- or blueberry-fed rats after 6 or 12 weeks of feeding.

	WC (n = 6)	WBB (n = 6)	SC (n = 6)	SBB (n = 6)
**Urinary NOx** (umol/mg creatinine)				
**6 weeks**	1.16±0.18#	0.57±0.10	2.01±0.27* $	0.97±0.13#
**12 weeks**	1.08±0.15#	0.62±0.12	3.85±0.68* $	2.09±0.31#
**Cortical NOx** (umol/mg protein)				
**6 weeks**	1.18±0.08#	1.34±0.14	1.92±0.17*	1.80±0.11
**12 weeks**	1.40±0.09#	1.09±0.06	2.29±0.22* $	1.33±0.18#
**Medullary NOx** (umol/mg protein)				
**6 weeks**	1.57±0.21#	1.66±0.17	3.08±0.53* $	1.41±0.18#
**12 weeks**	1.82±0.28#	1.84±0.23	4.78±0.46* $	1.97±0.20#

Abbreviations used: WC  =  WKY corn-fed, WBB  =  WKY blueberry-fed, SC  =  SHRSP corn fed, SBB  =  SHRSP blueberry-fed, NOx  =  nitrate/nitrite.

*p≤0.05 vs. WC;

# p≤0.05 vs. SC;

$ p≤0.05 vs. SBB.

Our results from the 2-day feeding study indicate that a hormetic effect of BB may indeed exist in the prevention of hypertension-induced renal hemodynamic alterations. The ‘xenohormesis’ hypothesis proposes that animal species have evolved the ability to use chemical cues from plant species to mount a preemptive defense response that increases its chances of survival [16],[35]. Polyphenols, among other phytochemicals, are thought to exert many of their beneficial effects via hormetic mechanisms [35]. In contrast to the clear evidence of reduced RONS production in the long-term studies, results from the 2-day feeding study indicated significant increases in total ROS, superoxide, and peroxynitrite production in kidney, brain, and liver tissues of BB-fed rats when compared to corn-fed rats. As a response to this situation, increased catalase activities were found with 2-day BB feeding, but only in kidney cortex and left ventricular tissues. Overall, the EPR and antioxidant assay results suggest that, in the case of BB feeding, an initial oxidative stimulus is produced, which is presumably required for the antioxidant defense to be activated, thereby supporting the assertion that a hormetic effect is involved in the protection afforded by BB *in vivo*. Since we evaluated hormetic responses only at the 2-day time-point of BB exposure, further analysis is required to document in detail the kinetics of ROS production and antioxidant responses.

In summary, our experiments examining rats chronically maintained on a BB-enriched diet for 6 or 12 weeks found preservation of renal hemodynamics and decreased blood pressure. Further, the BB diet decreased RONS production and preserved the status of some endogenous antioxidant systems in the kidney cortex and medulla of chronically fed hypertensive rats. The beneficial effects of the BB diet may be due to a hormetic effect, as evidenced by our results from the 2-day feeding experiment, where RONS production was increased in all tissues of BB-fed animals, while responses of the catalase and glutathione systems were in a state of flux, with some systems elevated at that time-point and others unresponsive. While the current results indicate major therapeutic benefits of the BB diet on renal function and pathology, it should be acknowledged that the effects reported pertain to prevention of the pathogenesis rather than treatment of the condition. Additional experiments will need to be conducted to assess treatment potential. In support of this possibility, the same BB diet has been shown to both confer protection against myocardial ischemia when started before the occurrence of myocardial infarction [13] as well as protection from further myocardial dysfunction when started two weeks after myocardial infarction [36]. These results in the myocardium indicate the potential for BB to be used as a preventative and as a treatment. We are planning follow-up experiments in this regard. This is the first demonstration, to our knowledge, of the effectiveness of a readily available natural product in an acceptable, consumable quantity to significantly attenuate hypertension-induced renal functional alterations.
